# Antiandrogenic and antimineralocorticoid health benefits of COC containing newer progestogens: dienogest and drospirenone

**DOI:** 10.18632/oncotarget.19833

**Published:** 2017-08-03

**Authors:** Pedro-Antonio Regidor, Adolf E. Schindler

**Affiliations:** ^1^ Exeltis West Europe & Germany, Ismaning, Germany; ^2^ Institut für Medizinische Forschung, Essen, Germany

**Keywords:** dienogest, drospirenone, therapeutics, antiandrogenic, antimineralocorticoid

## Abstract

Data have demonstrated that COCs, besides offering a satisfactory and safe contraception, offer a variety of non-contraceptive health benefits and therapeutic positive aspects. Many prescribes and users, however, do not realize these positive aspects especially the non-contraceptive health benefits. While the contraceptive use is the primary indication for COC use for most women, these users should be advised in regard of the non-contraceptive benefits when contraception is discussed and prescribed.

Using COCs specifically for non-contraceptive indications is an off-label use in many clinical situations (only some exceptions as e.g. acne vulgaris in some countries are allowed clinical entities for the use of these drugs). Therefore, appropriate discussions with the patient regarding this fact should performed and documented by the prescribing physicians.

Independent of the off-label situation, COCs containing the newer progestogens dienogest and drospirenone with their antiandrogenic and antimineralocorticoid health benefits play an important role in the management of many diseases and their use should therefore be considered by clinician’s.

This review will focus on the effects of these COCs on the endometrium, the skin, the fat tissue and the premenstrual syndrome.

## INTRODUCTION

Since starting the use of oral hormonal contraceptives as combined estrogen/progestogen drugs in 1960, COC have experienced a continuous change in used progestogens showing changing aspects of the partial effects in addition to its inhibition of ovulation [1]. These aspects of progestogen actions have been shown to be useful in incorporating aspects of non-contraceptive use into the possibilities of therapeutic uses creating a wide range of positive effects besides the primary use as contraceptives. During the last decades also changes in regard of the used estrogens have been implemented [1]. A general awareness to the non-contraceptive benefits of hormonal contraceptives has to be reached, as these drugs, besides their objective high efficacy and safety have been shown to have non-contraceptive benefits. In addition to their clinical value in different medical indications, COC´s have a very favorable cost/benefit ratio and a good level of compliance in comparison to other drugs used for medical indications. It has been stated that the non-contraceptive health benefits of COC´s represent an important aspect of the overall impact of this group of drugs beyond their primary use [2].

It is also important to point out that:

Thirteen percent of women aged between 15 and 19 years become pregnant voluntarily or not each year, a ratio that has not changed statistically since the 70s [3].

Approximately eighty-five percent of the above-mentioned pregnancies are unintended [4]. The economic and social impact factors of the one million teenage pregnancies each year in USA represent an important political factor [4].

Avoiding unintended pregnancies is a main concern of most sexually active women, especially the adolescents. It is estimated that in the year 1995 eighty-one percent of the women aged between 15 and 19 years at risk of unintended pregnancy were using contraceptive methods; many of them reported using two methods—one to avoid sexual transmitted diseases (STDs) and the second as the contraceptive one. The most used contraceptive methods were either oral contraceptives (44% of the cases), male condoms (46% of the cases) or in 8% both systems. However, the success with these methods depends heavily on user compliance. Especially with adolescents the problems are the incorrect intake of the tablets or failure to use condom just before sexual intercourse. These are USA data, but this topic is similar in almost all countries of the world [5].

These data emphasize the need of an effective contraception. The newer progestogens dienogest and drospirenone in combination with estrogens are therefore useful tools in the management of both; effective contraception to avoid unintended pregnancies and by the same way to offer non-contraceptive health benefits. Table 1 depicts the partial effect patterns of these two progestogens and of others.

**Table 1:** Partial activities of different progestogens. From Regidor [40].

	Progestogen	Anti- gonadotrop	Anti- estrogen	Estrogen	Androgen	Anti- androgen	Gluco- corticoid	Anti- mineralo corticoid	Pro Coagulatory
**Progesterone**	**+**	**+**	**+**	**-**	**-**	**+/-**	**+**	**+**	**-**
**Dydrogesterone**	**+**	**-**	**+**	**-**	**-**	**+/-**	**-**	**+/-**	**-**
**Medrogestone**	**+**	**+**	**+**	**-**	**-**	**+/-**	**-**	**-**	
**17a-Hydroxy-Progesterone Derivates**									
**Chlormadinonacetate**	**+**	**+**	**+**	**-**	**-**	**+**	**+**	**-**	**-**
**Cyproteronacetate**	**+**	**+**	**+**	**-**	**-**	**++**	**+**	**-**	**-**
**Megestrolacetate**	**+**	**+**	**+**	**-**	**+/-**	**+**	**+**	**-**	**+**
**Medroxyprogesteroneacetate**	**+**	**+**	**+**	**-**	**+/-**	**-**	**+**	**-**	
**19-Nor-Progesteron-Derivates**									
**Nomegestrolacetate**	**+**	**+**	**+**	**-**	**-**	**+/-**	**-**	**-**	**-**
**Promegeston**	**+**	**+**	**+**	**-**	**-**	**-**	**-**	**-**	**-**
**Trimegeston**	**+**	**+**	**+**	**-**	**-**	**+/-**	**-**	**+/-**	**-**
**19-Nortestosterone-Derivates**									
**Norethisterone**	**+**	**+**	**+**	**+**	**+**	**-**	**-**	**-**	**+**
**Lynestrenol**	**+**	**+**	**+**	**+**	**+**	**-**	**-**	**-**	**-**
**Norethinodrel**	**+/-**	**+**	**+**	**+**	**+**	**-**	**-**	**-**	**-**
**Levonorgestrel**	**+**	**+**	**+**	**-**	**+**	**-**	**-**	**-**	**-**
**Norgestimate**	**+**	**+**	**+**	**-**	**+**	**-**	**-**	**-**	**-**
**Desogestrel**	**+**	**+**	**+**	**-**	**+**	**-**	**-**	**-**	**-**
**Gestoden**	**+**	**+**	**+**	**-**	**+**	**-**	**+**	**+**	**-**
**Dienogest**	**+**	**+**	**+/-**	**+/-**	**-**	**+**	**-**	**-**	**-**
**Spirolonactone Derivate**									
**Drospirenone**	**+**	**+**	**+**	**-**	**-**	**+**	**-**	**+**	**-**

By the same way every prescription should also take into consideration the potential risks associated with the use of COC; e.g. the occurrence of thromboembolic events.

These risks still remain very low and are under continuous evaluation. The last referrals of the European Medicine Agency of 2014 [6] rated the risk for the use of COC with drospirenone at 9-12 cases under 10.000 users (0,1 %) and declared 2017 [7] that this risk for dienogest containing COC with ethynyl estradiol is still unknown and may be lower than that of other progestogens in combined formulations.

## HISTORICAL DEVELOPMENT OF PROGESTOGENS

In 1951 C. Djerassi and L. Miramontes converted 3-methoxy-estradiol into a 19-nortestosterone derivate with the help of the Birch reduction. In the next steps this 19-nortestosterone derivate was subsequently transformed by means of several chemical steps into 17α-ethinyl-19- nortestosterone (norethisterone) [8]. Norethisterone progestogenic potency was about 20-fold higher than that of ethisterone. 19-norprogesterone was also synthesized in 1951 by G. Rosenkranz and C. Djerassi using the same chemical method (Birch reduction). This substance was orally inactive, but it represented a potent progestogen after parenteral administration [8]. This progestogen is the basic substance of a series of 19-nor progesterone derivatives that have been applied till the beginning of this century (e.g., norhydroxyprogesterone caproate) or are still used today for contraception and/or hormone therapy as efficient progestogens (e.g., trimegestone, segesterone acetate, NOMAC).

Schering Germany developed due to the work of Junkmann and Schenk in 1951 norethisterone acetate. F. Colton synthesized norethynodrel at the G. D. Searle company from Chicago, Illinois. Afterwards dimethisterone that was developed in 1957 in England. The first use of this relatively weak progestogen was contraception, especially in sequential oral contraceptives [9].

Dimethisterone, like other progestogens, disappeared from the market. It was also Junkermann who developed 1954 at Schering the first progesterone derivative: 17α-acetoxyprogesterone. Medroxyprogesterone acetate, megestrol acetate and chlormadinone acetate followed in the years 1957, 1957 and 1959 respectively (all at Syntex). The prodrugs of norethisterone lynestrenol and ethynodioltediacetat like norethynodrel, and D, L-norgestrel were synthesized in the 1960s [9].

P. Duphar developed in 1959 the retroprogesterone derivative dydrogesterone (Philips-Duphar); in 1961 cyproterone acetate was synthesized by R. Wiechert at Schering. Desogestrel 1972 at Organon and dienogest 1978 by Hübner and Ponsold at Jenapharm [9].

Wiechert and collaborators synthesized in 1976 drospirenone at Schering AG. However, it took about 25 years, until its pharmacological potential was detected and the drug brought into the market in the year 2000 [10]. Table 2 depicts the different progestogen groups in relation to their chemical structure and year of development.

**Table 2:** Structural groups of progestogens and the year of development.

Structurel Groups		Progestogen	Year of synthetization and/or market introduction
Progesterone		Progesterone	1933/1997
Retroprogesterone		Dydrogesterone	1959
Progesterone Derivate		Medrogestone	1964
17a-Hydroxyprogesterone Derivates	Pregnanes (C21)	Medroxyprogesterone acetate	1957
		Megestrol	1959
		Chlormadinone acetate	1959
		Cyproteroneacetate	1961
17a-Hydroxynorprogesterone Derivates	Norpregnanes (C20)	Nomegestrol acetate	1986
		Gestenoron Caproate	1973
19-Norprogesterone Derivates	Norpregnanes (C19)	Demegestone	1974
		Promegestone	1983
		Nestorone	2001
		Trimegestone	2001
19-Nortestosterone Derivates	Estranes (C18)	Norethindrone	1951
		Norethisterone acetate	1951
		Lynestrenol	1961
		Norethinodrel	1957
		Ethynodiol Acetat	1967
19-Nortestosterone Derivates	Gonanes (C17)	Norgestrel	1966
		Levonorgestrel	1966
		Desogestrel	1981
		Etonogestrel	1998
		Gestoden	1986
		Norgestimate	1986
		Dienogest	1978
Spirolonactone Derivate		Drospirenone	1976

## STRUCTURE, ACTIVITY AND METABOLISM OF DIENOGEST AND DROSPIRENONE

### Dienogest

**Figure 1:**
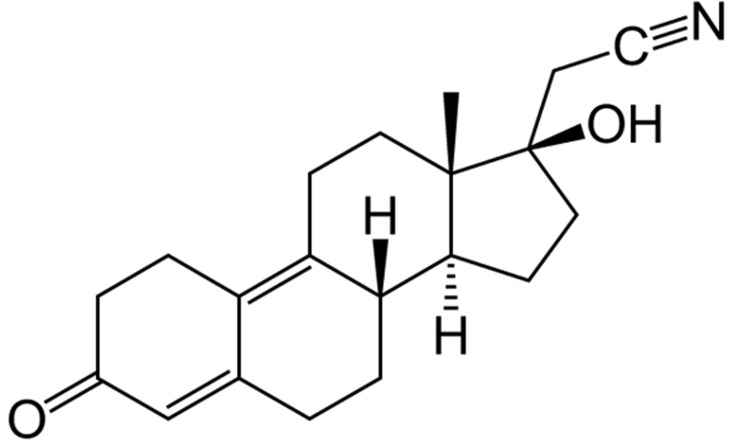
Chemical structure of the progestogen dienogest

The hormonal pattern and structure of DNG (see Figure 1) is different from that of other derivatives from nortestosterone as far as no ethynyl group is positioned at the C17α domain. Instead of it a cyanomethyl group is part of this drug (Figure 1). It is known that irreversible inhibition of CYP enzymes result from ethinylated steroids through the oxidatively activated ethinyl group. This lack in Dienogest leads to its non-action on CYP enzymes [11]. CYP enzymes regulate the ovarian steroid biosynthesis and the inactivation of own or external steroid hormones, progestogens with ethinylated groups - so as ethynyl estradiol - may directly impair follicular activity and inhibit follicular degradation. This is one model approach to understand why the other nortestosterone derivatives have a pharmacological low dose when compared to dienogest.

DNG is the only nortestosterone derivative with no androgenic, but an antiandrogenic potency. Depending on the used tests it´s antiandrogenic activity is about 30% of that of cyproterone acetate. Dienogest has a relatively low binding affinity to the progesterone receptor and on the other site dienogest nevertheless shows a very strong progestogenic effect on the endometrium. Dienogest has with about 6mg a very similar transformation dose per cycle to that of levonorgestrel. The reason for this is the low binding affinity of dienogest to SHBG and CBG. Only 10 % is bond to these proteins so that high serum levels are achieved and subsequently high concentrations in cells. Dienogest has no glucocorticoid, antimineralocorticoid or estrogenic partial activity, so that estrogen-induced positive changes of certain hepatic serum proteins are not counteracted [11].

Dienogest is absorbed rapidly and has a bioavailability of about 95%, with a short elimination half-life of approximately 9 hours. After a single oral administration of 2 mg DNG and 30 μg EE, maximum levels of 53 ng/ml dienogest are reached within 2 hours. Afterwards a fast decline to 7 ng/ml after 24 hours is observed [11]. Dienogest is degraded and metabolized through the reduction of the Δ4-3-keto group, through hydroxylation reactions and hereby the elimination of the cyano group.

### Drospirenone

**Figure 2:**
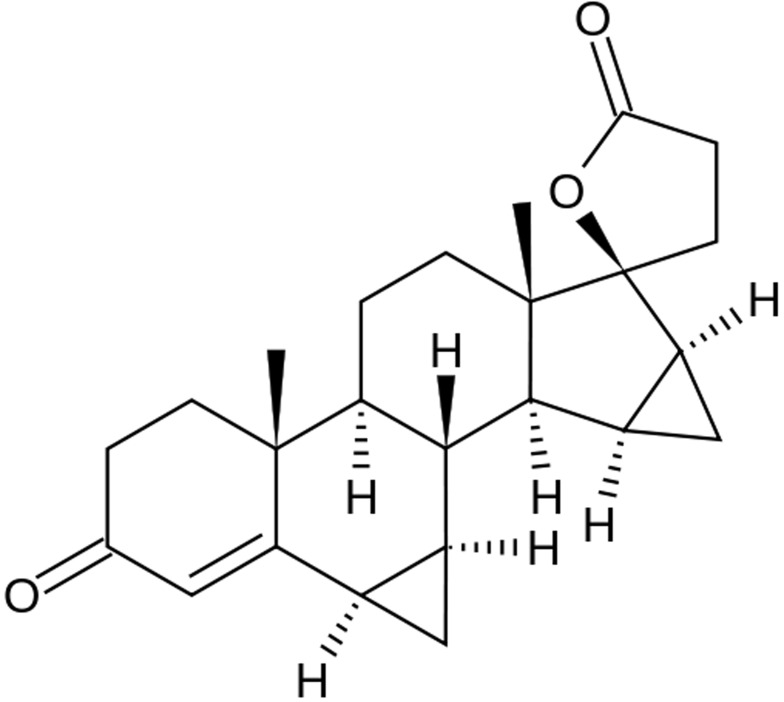
Chemical structure of the progestogen drospirenone

Drospirenone, a derivative of 17α-spirolactone, has a similar chemical structure to the aldosterone antagonist spironolactone (see Figure 2). It has a low to moderate binding capacity to the PR, excellent binding properties to the mineralocorticoid receptor and a low binding affinity to the androgen receptor [12]. Drospirenone has, in relation, only 10% of the progestogenic activity of levonorgestrel on the human endometrium. When used for hormonal replacement treatment doses of 3 mg per day have to be used. Due to the strong antimineralocorticoid effect of drospirenone the use of 2 mg in fertile women during the follicular phase caused an increase in sodium excretion. By the same the way a rise in the plasma renin activity by 100% was observed, so that the effect of sodium excretion was compensated. The aldosterone serum levels raised by 65% [13]. DRSP has also a antiandrogenic activity of that in a range of 30% of that of cyproterone acetate. Similar to dienogest it has no estrogenic and no significant glucocorticoid activity [14].

Also, similar to dienogest DRSP has no binding affinity to SHBG and CBG because it is bound in the serum to albumin so that the free blood amount is about 3 to 5%. The oral bioavailability is in a range between 85 and 75 %.

After a single administration of 3 mg drospirenone in combined oral contraceptives serum levels of 35 ng/ml can be measured after one to two hours of intake. After this peak, the levels go done, but 24 h later the DRSP concentrations in the serum remains at values of 20 to 25 ng/ml. This is the reason why a accumulation of drospirenone in blood after repeated dosing, and treatment in combination with estrogens leads to peak serum concentrations of 60 ng/ml after seven to ten days. DRSP half-lives are 1.6 hours (t1/2α) and 27 hours (t1/2β). Drospirenone is depleted through a metabolic pathway that consist in the opening of the lactone ring resulting in an acid group. Afterwards a reduction of the Δ4-double bond is performed [14]. In HRT treatment dosages with 1 mg estradiol and 1, 2 or 3 mg DRSP given continuously provided an efficient and safe protection of the endometrium, it leads to an improvement of menopausal symptoms and caused a significant increase of the bone mineral density. After one year of use 80 % of the women experienced a continuous amenorrhea [15]. As DRSP has no partial androgenic properties, no counteraction to the estrogen-induced positive actions of the lipid metabolism could be observed. DRSP in these formulations caused a slight blood pressure lowering effect that is not different to that of other formulations containing estradiol and progestogens [15].

## TREATMENT, PREVENTION, AND DISEASE RISK REDUCTION DUE TO THE ANTIPROLIFERATIVE AND ANTIANDROGENIC PROPERTIES OF DIENOGEST (=DNG) IN COMBINED HORMONAL CONTRACEPTIVES

### Efficacy

The overall pearl index of the combined ethinylestradiol/dienogest contraceptive is 0,21 [16].

Dienogest and menstrual bleeding disorders such as poly- and/or oligomenorrhoea, hypermenorrhea/menorrhagia

The most important physiological effect of progestogens on the endometrium is to perform a secretory transformation after the estrogen-stimulated proliferation

With the decreasing hormone secretion in the wake of the corpus luteum regression, at the end of the ovarian cycle it comes to menstrual bleeding in the sense of a withdrawal bleeding.

The long-term use of progestogens, however, cause an atrophy of the endometrium.

The most important reason for the use of dienogest in all of its clinical applications and especially in the treatment of endometriosis is its extremely high progestogen effect on the uterine endometrium.

The safety of dienogest on the endometrium has been documented in various experimental investigations [17] and clinical studies [18, 19].

The determination of the relationship between ovulation inhibiting dose (mg/day) to the endometrial transformation dose (mg/cycle) in the Kaufmann test resulted in the extremely high value of 17 [17] for dienogest.

The endocrinological pharmacodynamics of dienogest is therefore characterized by its peripheral effect especially on the endometrium. This effect is quite similar to the changes after the action of progesterone.

Individual trials [20, 21] could demonstrate that the frequency of dysmenorrhea when using a combined oral contraceptive with ethinylestradiol/dienogest was 28.8% prior to application, 12.9% in the 1st application cycle and close to zero after the 6th cycle of treatment.

A good cycle control was also reported in a post-marketing surveillance. Spotting’s and breakthrough bleedings occurred during the first cycle of application in 5.0% and 3.4% of the women, respectively. Silent menstruations occurred in 2.0% of the cycles or throughout the entire application in 5.9% of women [21].

This corresponds to the frequencies of withdrawal bleedings in the phase III study of Moore et al. (ethinylestradiol/dienogest) (3% of cycles) [22] and in the data collected by Golbs [23, 24] (4.8% and 3.7% during the 1st cycle, in the other cycles about 3%).

### Dienogest and acne

Acne is a multifactorial, inflammatory disease of the sebaceous follicles.

Androgens play a pathogenetically important role due to its stimulating effects on the sebaceous gland activity and epithelial proliferation in the area of the sebaceous gland duct and the acroinfundibulum of the follicles.

Acne can represent a severe impairment in the quality of life for those affected, coupled with fear of social exclusion and till the development of depression [25].

Ethinylestradiol/dienogest has a demonstrable favorable impact on an existing moderate acne due to its antiandrogenic effect of dienogest in women. This combination is approved in some countries with a specific legal use in the management of this disease.

In a controlled multinational, multicenter, double-blinded randomized study 1 338 women with a light to moderate acne papulopustulosa of the face (16-45 years old) were treated with different combined oral contraceptives. N = 525 with 6 cycles with ethinylestradiol/dienogest, ethinylestradiol/cyproterone acetate was used in n = 537,264 women received a placebo [26].

With ethinylestradiol/dienogest the symptoms improved significantly compared to the placebo group, based on the number of inflammatory lesions (papules, pustules and nodules) and the total number of lesions.

In its efficacy, the combination of ethinylestradiol/dienogest was equal to the comparison drug with contained cyproterone acetate and ethinylestradiol.

A definitive improvement of face acne - evaluated on the basis of the 6-step scale of IGA (investigator global assessment)- was observed with ethinylestradiol/dienogest in 91.9% of the women and in group with ethinylestradiol/cyproterone acetate in 90.2% of the cases and in the placebo group in 76.2% of the women [26].

A further investigation of 6004 women who had acne vulgaris revealed that in the course of a treatment with ethinylestradiol/dienogest for the duration of 6 cycles in 29% of cases a cure of acne was described. In 61% of the women an improvement of acne was seen. Even women with a severe form of acne experienced a benefit in more than 90% of the cases [27].

Others:

Many other beneficial effects beyond the above mentioned have been described.

They are listened in Table 3.

**Table 3:** Non-hormonal benefits of combined hormonal contraceptives with the progestogens dienogest and drospirenone Modified from Schindler [41].

1.) Menstrual bleeding disorders
2.) Dysmenorrhea
3.) Premenstrual syndrome/premenstrual dysphoric disorders
4.) Signs of androgenization
5.) Ovarian cysts
6.) Pelvic inflammatory diseases (PID)
7.) Rheumatoid arthritis
8.) Preservation of bone density
9.) Endometriosis/adenomyosis
10.) Uterine myoma
11.) Benign breast diseases
12.) Ovarian cancer
13.) Endometrial cancer
14.) Colon cancer

## TREATMENT, PREVENTION, AND DISEASE RISK REDUCTION DUE TO THE ANTIMINERALOCORTICOID AND ANTIANDROGENIC PROPERTIES OF DROSPIRENONE (=DRSP) IN COMBINED HORMONAL CONTRACEPTIVES

### Efficacy

The overall pearl index of the combined ethinylestradiol/drospirenone contraceptive is 0,64 even when used in flexible cycles [28].

### Drospirenone (DRSP) and acne

Eight hundred and eighty-nine women with an age between 14 to 45 years, that suffered from a moderate acne were treated in two multicenter, double blind, randomized, placebo-controlled studies with a combination of ethinylestradiol/drospirenone or placebo for a period of time of six months in 28 day cycles. The first primary efficacy endpoints were the percentage in the change of the inflammatory, the non-inflammatory and the total lesions. The second primary endpoint was the amount of women with a “clear” or “almost clear” rating on the Investigator’s Static Global Assessment (ISGA) scale on day 15 of cycle 6. Maloney et al. [29] could show an improvement in 21 % in ISGA success, a 46 % reduction of total lesions, a 51% reduction of inflammatory lesions and a 42 % reduction of noninflammatory lesions in the drospirenone group of patients. In the Koltun et al. [30] study the values were 15%, 42 %, 48% and 39 % respectively.

### Premenstrual syndrome (PMS)

The premenstrual symptoms can be considered as an individual persistent problem among women during their reproductive life. The classical periodically appearing medical disorders have an impact on physical, emotional, and psychological conditions influencing adversely the quality of life of the affected women and in many times also the surrounding individuals. Several studies have tried to develop new drugs to alleviate the effects of the symptoms. Wichianpitaya et al. [31] could show a significant improvement in the mean Women´s Health Assessment Questionnaire (WHAQ) scores. This score included water retention, impaired concentration, increased appetite, feeling of wellbeing, not desirable hair changes, negative effects and a total score and it was measured at three times during the menstrual cycle (premenstrual, menstrual and postmenstrual). All the parameters improved significantly after 6 months of treatment with a combination of ethinylestradiol/drospirenone for all the parameters and in at times of the menstrual cycle.

This is due to the fact, that drospirenone and progesterone are very close progestogens regarding their mode of action. Progesterone has a non-genomic pharmacological way of action that causes sedative effect on the central nervous system: This could be demonstrated in the down regulation of the CNS activity measured by electroencephalograms and the changes in the arousal threshold stimuli in the hypothalamus of various animals. Other neurological conditions such as an increase of the nervous excitability, irritability, tension, anxiety, and/or aggression are due to a low amount of progesterone in the blood serum [15]. The possible reasons of the benefits DRSP on the central nervous system may be since the chemical structure of DRSP is very like that of progesterone. These effects on the CNS are more pronounced in comparison to other progestogens [32]. Some investigations could show abnormally elevated plasma testosterone levels in women with premenstrual symptoms with concomitant signs of aggressiveness [33]. It can be postulated that the improvement of the premenstrual symptoms in women who receiving DRSP in comparison to those using other progestogens was related to the antiandrogen potency of DRSP. Considering that DRSP exerts no binding affinity to the Sex Hormone Binding Globulin (SHBG), DRSP per se doesn´t lead to a rising of serum-free testosterone that increases the symptoms of the premenstrual symptoms including the aggressiveness. DRSP does not counteract the effects of EE on the increasing levels of SHBG [15].

### Drospirenone and fat tissue

Experimental data could show the positive effects of drospirenone of the remodeling of fat tissue. These data support the neutral effect of drospirenone on the body weight of female users and the utility in overweight patients with a PCOS.

Rezk et al. [34] could show in a prospective, case-control study over a period of three years on 202 overweight and7or obese women older than 35 years, who received ethinylestradiol/drospirenone for 36 cycles or whom an intrauterine device was inserted the following results. Both groups did not differ in regard to body weight, waist circumference, blood pressure and fasting blood glucose levels (p > 0.05). A statistically significant difference could be observed in favor of the EE/DRSP group when analyzing triglycerides, total and LDL and HDL cholesterol after 24 and 36 cycles of use (p < 0.05).

The basis of these findings can be seen in the influence of drospirenone on the mineralocorticoid receptor. The group of Armani et al. [35] could show that the mineralocorticoid receptor (MR) controls the adipocyte function.

They investigated responses to the mineralocorticoid receptor antagonists spironolactone (20 mg/kg/d) and drospirenone (6 mg/kg/d) in C57BL/6 mice: These animals were fed with a high-fat diet for 90 days. DRSP and spironolactone improved the high-fat diet-induced impairment in glucose tolerance, and prevented a body weight gain and the growth of white fat tissue. Notably, either MR antagonist induced up-regulation of brown adipocyte-specific transcripts and markedly increased protein levels of uncoupling protein 1 (UCP1) in visceral and inguinal fat depots when compared with the HF diet group.

Hence, they could show a rationale for the use of MR antagonists to prevent the adverse metabolic consequences of adipocyte dysfunction. Mineralocorticoid receptor antagonism induces browning of white adipose tissue through impairment of autophagy and prevented adipocyte dysfunction in high-fat-diet-fed mice.

21, 24 day or continuous intake of DRSP containing COC

As the pearl index between all three day intake formulations in combined oral contraceptives is almost similar, COC with the lowest ethinyl estradiol dosage and the lowest amount of drug day intake should be used to achieve the lowest side effect profile; especially the thromboembolic risks [36, 37]. For those women with pms and/or pms better non contraceptive results will be obtained with the 24 day regime [38] and also for those with very prolonged menstrual cycles with late ovulations (after day 20 or more) before starting the use of COC to improve the contraceptive efficacy. Here continuous cycles should also be considered [39].

Others:

Many other effects beyond the above mentioned have been described.

They are listened in Table 3.

## CONCLUSIONS

The new progestogens Drospirenone (DRSP) and Dienogest (DNG) offer in different combinations with 20 or 30 µg ethinylestradiol a splendid opportunity for the individual needs of adolescents especially in regard to the high antiandrogenic and antimineralocorticoid effects not only for contraception but also for different non-contraceptive benefits (see Table 1). The primary need of a good and effective contraception with pearl indices lower than 0,65 enhances the approach to individualized contraception going away from contraceptives with androgenetic partial effects moving towards progestogens with antiandrogenic and/or antimineralocorticoid partial activities.

Compliance with ethical Standards:

### Ethical approval

This article does not contain any studies with human participants or animals performed by any of the authors.
